# Memoir on the Hybridity of Animals

**Published:** 1859-11

**Authors:** Anthony Peniston

**Affiliations:** Prof. of Physiology in the New Orleans School of Medicine


*Memoir on the Hybridity of Animals.* By ANTHONY PENISTON, M. D., Prof. of Physiology in the New Orleans School of Medicine.

Hybridity is the result of the crossing of different species of animals, and as the fruits of these ill-assorted unions, from the Minotaur downwards, have been considered sterile and incapable of propagating themselves, this negative quality has long been assumed as the best test of difference between animals, warranting therefore, their classification under different species. It is probable that the familiar instance of the mule, or the result of the cross between the ass and the mare, has had much influence in accrediting this opinion. Later investigations, however, have shown that no rule can be laid down *a priori*, on this subject, and that the universal sterility of hybrids is about as correct as the Ptolemeian system of astronomy. The fact is that the sterility of hybrids has been taken for granted without sufficient proof, pro or con, and is certainly not an infallible test of distinction between animals. No one would contend that the goat and the sheep do not belong to different species, yet it is now an established fact that the offspring of the union between the buck-goat and the ewe is fruitful. Recent experiments have shown that several other hybrids are also fruitful. Indeed, this subject has many practical as well as scientific applications; and we propose, in the following pages, to set forth some facts which have been recently published in Paris.

We are indebted for these views and opinions to our learned confrere, Doctor Paul Broca, of Paris; to whom we have already expressed our obligations in the preceding number in the memoir on the varieties of mankind. In the last number of the "Journal de Physiologie," which has just reached us, we have found the sequel of this interesting subject, and shall endeavor to present a bare abstract of this article for the benefit of our readers.

The result of the crossings between animals of different species can only be demonstrated by experiment, and no rule can be laid down, *a priori*, on this subject.

Thus it is that an animal may have sexual intercourse with an animal of a different species, though they may not procreate. On the other hand, the male of one kind may procreate with a female of another species when a male of the latter would not impregnate a female of the former, or it may be that both sides are equally fruitful. We might term the former kind unilateral hybridity, the latter liberal hybridity.

Thus, says Buffon, experiment has shown that the result of the union between the male of the goat and the female of the sheep is fruitful, but the union of the ram and the female of the goat is not fruitful.

This, however, is one of the few cases of unilateral hybridity; it more generally happens that both crossings are fruitful, though one may be uniformly more productive than the other. Thus the mule, or the result of the crossing between the ass and the horse, is more easily obtained than the cross between the jinny and horse.

And, strange to say, it has been ascertained that the hybrids resulting from those races which are most easily crossed are themselves the least fruitful. Thus the mule is generally barren, but the result of the cross between the hare and the rabbit, is exceedingly prolific.

There is another singular property attendant upon the procreative qualities of hybrids, viz: that some which would be sterile with hybrids of the same degree and species are nevertheless fruitful when crossed with either of the parent species. It has also been observed that certain hybrids which are fruitful with each other, even at the first crossing, would nevertheless fail in perpetuating the race, unless renewed by fresh accessions from the parent race.

It appears, therefore, that many different races of animals are susceptible of having sexual intercourse with each other, though these unions, from some cause or other, are not necessarily fruitful, and if fruitful in the first degree, are not necessarily bound to propagate their newly created species; whereas, others, on the contrary, seem to have no limits to their fruitfulness. It must be bore in mind, however, that many experiments of the kind have failed on a small scale, because, from the want of fresh individuals of the same kind, the experimentors have kept on crossing a few individuals with each other, brothers with sisters, parent and offspring, until these incestuous unions have resulted in perfect degeneration and barrenness. A great many curious questions naturally present themselves on the subject of hybridity. It may be asked what is the physical explanation of unilateral hibridity, as in the case of the goat and the sheep? Does it depend upon the period of the gestation of either of the species, upon the conformation of the organs of generation, upon the similarity of the ovaries, or of the spermatooza, or upon the rank which the animals are supposed to hold in the hierarchy of races? Of late years no department of natural science has received more advancement than that of embryology, and yet how far are we from possessing any data upon which we could proceed to answer the above questions? Like the cause of the difference of sexes, their solution is yet hid by the veil of time.

It is true that under this subject of hybridity the mooted question of the distinction of races again springs up, and that many philosophers have denied either the validity of the experiments, or that the races thus crossed were really distinct from each other. Thus, says Pritchard, many persons yet doubt that the dog and the wolf are of distinct species; and John Hunter believed that the dog and the jackal were the same animal, the former being only modified under the influence of domesticity. Even Doctor Carpenter has asserted, that the dog may be nothing more than a tame wolf; but any one must have an immense amount of credulity to accept such an assertion in lieu of argument, for there are numerous specific differences between the wolf and the dog. The howl of the wolf cannot be compared to the bark of the dog, any more than the braying of the ass with the neighing of the horse. It is a well known fact that the wild dogs of South America, which have remained in a savage state for several hunded years, have retained all their characteristics, and are as far from becoming wolves as they were in the beginning; they continue to bark like the domesticated dog, showing that this is a natural and not an acquired quality. Moreover, the wolf has resisted every attempt to domesticate it, and nothing has ever been adduced to show that a wolf can be transformed into a dog, or a dog into a wolf, after however many successive generations.

We are, therefore, constrained to admit that the dog and the wolf are two distinct species of animals, and that their hybrids are fruitful in every degree; but Doctor Broca, in his interesting memoir, has also shown that the sterility of the mule has been taken for granted, perhaps too readily, though it cannot be denied that in this respect they are inferior to both of the parent races. We doubt not, however, that if it could be demonstrated to be to the interest of breeders to propagate any cross of the mule with either of the parent species, it would be found that the mule is not so barren as is commonly supposed. Besides the mule there are several other hybrids of the genus *horse*. Thus the zebra is productive both with the horse and the donkey. It is well known that there are two varieties of mules; one which is the issue of the jack and the mare; the other called by the French, bardeau, is the issue of the stallion and the jenny. These two varieties of mules have been known from the most remote antiquity, and Aristotle distinctly mentions them. The former kind is by far the most common, though the latter seems, for some reason or other, to be preferred in the kingdom of Naples. It is generally supposed that the bardeau, or the offspring of the jenny, is smaller and less docile than his cousin the mule; but this may be a popular prejudice, and it is very possible that if the same care was taken in importing enormous jennies, and crossing them with the largest stallions, as is done by the selecting at great cost the largest jacks to breed from, it might be that the much abused bardeau might contest the palm of size, as he certainly would carry off that of elegance of form from his over-grown and long-eared relative, the common mule.

In each of these varieties of hybrids the characteristics of the parents are still easily recognized. They resemble the dam in the body; the sire in the head and extremities. Thus in the mule we recognize the short, thick head of the jack, its long ears, bony legs, and thin, scanty tail; on the other hand, the bardeau has a narrow but long head, its ears are shorter, its legs more muscular, and its tail more bushy, like its sire, the horse. There are other peculiarities in this parallel between these two varieties of hybrids which are curious enough. Every one has observed on horses those thorny productions which are found on the inner side of the four extremities, midway between the knee and the shoulder. In the ass those excrescences are found on the anterior extremities alone, and they are smooth instead of being rough and hard as in the horse. Now in the two varieties of mules these excrescences have characteristics common to both sets of parents. Thus the number is always derived from the father; their nature from the mother. The bardeau has four excrescences like the father, but they are perfectly smooth like the mother; on the other hand the mule has only two, and they are rough.

Besides this, it has been shown that the orbital arch of the bardeau resembles that of the horse; in the mule, it resembles that of the donkey. The maxillary sinus of the horse is divided into two antra, which do not communicate, though they do in the donkey; the mule inherits this peculiarity from his father; in the bardeau they have not been examined, but they probably follow the law of resemblance to the sire. The larynx of the ass is much more complicated than that of the horse, being especially characterized by a deep depression of the thyroid cartilage, which has been described by Herissant under the name of the *drum*. This anatomical difference must have its physiological significance, and the braying of the ass is said to depend upon this peculiarity of the larynx. The mule which brays like its sire, has also the drum; but the other hybrid neighs like the horse, and has probably the same structure of the larynx. There are some other structural differences between the ass and the horse, though they are not as invariably transmitted by hereditary descent as the preceding. Thus the ass has five lumbar vertebræ, and the horse, six; some mules have been found to have five, and others six lumbar vertebræ.

The question of the sterility of the mule has been anatomically studied, without, as yet, throwing much light on the subject. It appears that both varieties of mules have all the necessary organs of generation. The females have their periods of heat, and the male emit a spermatic fluid, though much contestation seems to exist as to its generative qualities. The latest authorities, however, admit that they have found it to contain spermatozoa, though perhaps some mules are deficient in this respect. It is probable, on the other hand, that a mule of either sex might prove fruitful if crossed with the opposite sex of the parent species, and it is probable that in those cases in which it has happened, the credit of the doubtful paternity, from the more than suspected impotency of the mule, has been bestowed upon some neighboring donkey. It is evident, therefore, that except the experiment be conducted with scrupulous care, doubts would attach to the procreative powers of the male mule.—With the female, however, whatever be the sire, there could be no doubt in the case. The parturition of the mule has long ranked among fables, and like a crowing hen, supposed to be a presage of dire misfortune. It has now been completely divested of its supernatural signification, and is only rare because seldom attempted.

The female of the mule gets in heat every spring; its ovaries, which have been examined by Mr. de Nanzio, do not appear to differ from those of the mare or the jenny.—The ovule presents the discus proligerus, the zona pellucida, the vasicula, the vitellus, and the germinative spot. And yet it must be that this ovule, anatomically perfect, is deficient in some respects since it is but rarely fecundated. It has been observed that the mules are oftener fruitful in warm climates than in the temperate zone. The occurrence is very rare in France, and all the known cases have taken place in Spain and Italy, or under the tropics. It is said that in tropical Asia the mules frequently conceive; but as their parturition is exceedingly difficult, they and their offspring generally perish. It appears, moreover, that the Cæsarian section has been successfully resorted to in these cases. Though it cannot be denied that the fertility of the mule is exceptional, there are many authentic cases in which they have proved fruitful, and their progeny survived many years; one of these is known to have lived to a very old age, and to have done good service in the Neapolitan cavalry.

We have, in the instance already cited of the goat and the ewe another instance of hybridity, for these animals do not belong to the same genus, much less to the same species. Buffon, in his great work on Natural History, was the first to draw public attention to the cross between the goat and the sheep. In Europe, this variety of hybrid has not been cultivated, and, in fact, seldom attempted, except for the purpose of experiment. It appears, however, that in some parts of South America, and especially in Chili, the hybrid between the goat and the sheep is raised in large quantities. They are said to be twice as large as the ordinary sheep, and are covered with a species of wool which is very long and soft. These furs are exceedingly valuable, and much esteemed in South America, where they are used for many different purposes, such as carpets, bed-covers, saddle-ornaments, and a variety of other purposes. This race of hybrids is very fruitful with each other, and with the goat especially. The hybrids of the half-blood are not the best, the hair is long but rough and straight. This first generation is again crossed with the sheep, and their offspring furnish the most valuable fur; so that these hybrids are three-fourths sheep, and one-fourth goat. Yet these quadroons have a tendency, after three or four generations, to return to the goat-type; their wool or fur becomes very harsh and rough, and it is found necessary again to change the breed. For this purpose the females of the latter are crossed with the males of the half-breed, thus giving a hybrid which contains three-eighths of the goat, and five-eighths of the sheep. Mr. Broca observes that these hybrids are thus shown to be fruitful with each other and with the parent race, affording at the same time an example of unilateral hybridity, since the issue of the ram and the goat is totally unknown in South America.

The cross between the wolf and the dog is an example of bilateral hybridity, for the offspring are fruitful with each other and with both of the parent races. Besides these there are numerous other examples of hybridity; as, for instance, between the buffalo and cow, between the different species of camels, and between the lamas, the alpaccas and vigognias of South America—all of the latter being animals of distinct species, though their hybrids are perfectly fertile. We have yet to speak of another variety of hybrids, which are exceedingly curious, not only as another important step in the accumulative evidence gathered by Mr. Broca, with great learning and talent, in defence of his opinion, but as exceedingly interesting in a practical point of view, viz: as a means of supplying an excellent article of food.

The hybrid to which we refer is the result of the cross between the hare and the rabbit. Of late years this race has been cultivated with great success by Mr. Alfred Roux, a gentleman residing in Angoulême, France. Mr. Roux, who is said to be a very intelligent man, and president of an agricultural society in his province, has succeeded admirably in obtaining the cross between the rabbit and the hare. His experiments were not undertaken to disprove the unity of the human race, but in order to procure a race of animals which would have the size and delicacy of the hare united to the greater reproductive qualities of the rabbit, and his more economical appetite; questions evidently of immense practical importance to any one going into the business on a large scale. Indeed, they seem to have been perfectly solved, and Mr. Roux supplies the market of his native town with several thousands of these interesting and excellent hybrids. The weight of the French rabbit, one year old, is about six pounds. The wild hare weighs about eight pounds, but in captivity they rarely exceed six pounds. Mr. Roux's hybrids at a year old, weigh, commonly, from eight to ten pounds; several have been found to weigh as much as twelve and fourteen pounds, and one actually weighed sixteen pounds; it measured two feet four inches in length, and its skin, which was preserved, had a most beautiful fur.

But this splendid result was not obtained without cost and trouble. The hare and the rabbit are two distinct races of animals, which, in their natural condition, are perfectly antagonistic to each other. They do not differ as much anatomically, as they do by their instincts, tastes and habits.— The rabbit is a gregarious animal, the hare lives perfectly alone; the rabbit burrows in the ground or in hollow trees, where they breed and rear their young brood; the hare, on the contrary, lives above ground, and only conceals himself in the brush and shrubbery. The period of gestation in both cases is thirty days, but the hare only bears two or three times a year, and brings forth from two to four young; the rabbit, on the contrary, bears as often as eight times a year, and brings forth never less than four, and generally from six to eight young at a birth.

Everybody knows the rabbit is easily domesticated, and that the tame rabbits multiply in great abundance. But all attempts to domesticate the hare have failed, and they never breed in captivity; moreover, the hare and the rabbit are at war with each other; the hare, though larger, is generally driven away by the rabbit, and hunters are aware that they are never found together; in fact, the rabbits must be destroyed before the hares will multiply. Besides these differences, the flesh of the hare is red, that of the rabbit is white; and the two kinds of meat have a different taste and smell; the small intestines are longer in the rabbit than in the hare, but the latter has a longer cæcum, though the large intestine is shorter than the rabbit's. The color of the hare is of a brownish red, that of the rabbit an iron grey. These two animals inhabit, from time immemorial, the same climates and the same countries, and live upon the same food, yet they have ever remained perfectly distinct, and could never have had a common origin.

Buffon had made several unsuccessful attempts to cross the hare and the rabbit; so much so that he finally declared it impossible. Mr. Roux commenced his experiments in the year 1847, and three years afterwards had so far succeeded that he found it expedient to continue his operations on a large scale. Mr. Broca visited this establishment in 1857, and found there several generations of these hybrids; he says, that looking at the various intermediate types between the hare and the rabbit, and their different grades of color, he was reminded of the public promenades in Havana, where all shades of transition are found, from the pure white to the jet black. He again visited Mr. Roux, in 1859, and found his establishment in a very prosperous condition; the hybrids were larger and more beautiful than either of the parent races. In order to succeed in obtaining this cross-breed, the hare must be taken when very young, say two or three weeks old, and brought up in company with female rabbits, entirely separated from any males of their own species; then the hare gradually loses much of its savage nature, and the rabbits accept him as their natural lord; so that they are actually cheated into an unnatural marriage, though the bans would be soon broken if they discovered their mistake. Mr. Roux has not tried the converse experiment, or the union between the male rabbit and the female of the hare.

The rabbits, when breeding to the hare, generally bear from six to eight young. The hare is kept separated from the female rabbits, and they are only put together during one night when the latter are in heat. The hybrids of the half-blood have more resemblance to the hare than the rabbit; their fur has a slightly reddish tinge, but the grey of the rabbit evidently predominates; the ears are longer than those of the rabbit, and they are intermediate in size between the two parents. These hybrids are very fruitful with each other, and with either of the parent races. There is no peculiar advantage in propagating these half-breeds; for a better result is obtained by crossing them over again with the hare; and they become, therefore, quadroons. Unlike their namesakes in the human species, they resemble the rabbit more than the hare; whereas the beautiful quadroon resembles more her ancestors of European origin, than her Congo grand-mother. These quadroon rabbits, however, are not very prolific, they do not bear more than from two to five young, approximating to the hare in this respect, though otherwise resembling the rabbit; on this account Mr. Roux crosses the quadroon with the half-breed, and obtains an animal which is three-eighths rabbit and five-eights hare. These are quite as large as the quadroon, and far more prolific; they grow very fast and have more flesh than any other variety. The ordinary rabbit is worth about a franc in the market of Angoulême; Mr. Roux's three-eighths hybrids command readily double the price at four months.—The flesh of these animals is darker in color than that of the rabbit, and far more delicate to the taste, having, it is said, some resemblance to that of the turkey. Altogether they are a very interesting quadruped, and we hope some enterprising man will imitate Mr. Roux's example on this side of the water. A variety of the hare exists in great abundance in Texas; it is an exceedingly wild, but large and beautiful animal, to which the French hare is a perfect pigmy. If a cross could be obtained between these and the rabbit, we should have a race of giants, the produce of which would be worthy the great State whence they came.

Such are some of the curious facts connected with the subject of hybridity; and, in giving them to our readers, we have been more prompted by their utilitarian point of view than by their importance in support of the great dogma they unequivocally maintain. At all events, it will appear that, as with a great many other physiological questions, we are still on the threshhold of the science, and we may, at an early day, stumble upon some facts which shall overthrow all the past. So far, we are not able to generalize upon the subject; for neither the proximity of the species, nor their instincts, nor their mode of life, nor their structure, nor their period of gestation, allow us to draw any infereuce as to the fruitfulness or sterility of their union. But we look forth with impatience to the conclusion of Doctor Broca's article on the hybrids of the *genus homo.—N. O. Medical News and Hospital Gazette.*

